# Unraveling a Cavity-Induced Molecular Polarization Mechanism from Collective Vibrational Strong Coupling

**DOI:** 10.1021/acs.jpclett.4c00913

**Published:** 2024-05-08

**Authors:** Dominik Sidler, Thomas Schnappinger, Anatoly Obzhirov, Michael Ruggenthaler, Markus Kowalewski, Angel Rubio

**Affiliations:** † Laboratory for Materials Simulations, Paul Scherrer Institute, 5232 Villigen PSI, Switzerland; ‡ Max Planck Institute for the Structure and Dynamics of Matter and Center for Free-Electron Laser Science, Luruper Chaussee 149, 22761 Hamburg, Germany; § The Hamburg Center for Ultrafast Imaging, Luruper Chaussee 149, 22761 Hamburg, Germany; ∥ Department of Physics, Stockholm University, AlbaNova University Center, SE-106 91 Stockholm, Sweden; ⊥ Center for Computational Quantum Physics, Flatiron Institute, 162 Fifth Avenue, New York, New York 10010, United States; # Nano-Bio Spectroscopy Group, University of the Basque Country (UPV/EHU), 20018 San Sebastián, Spain

## Abstract

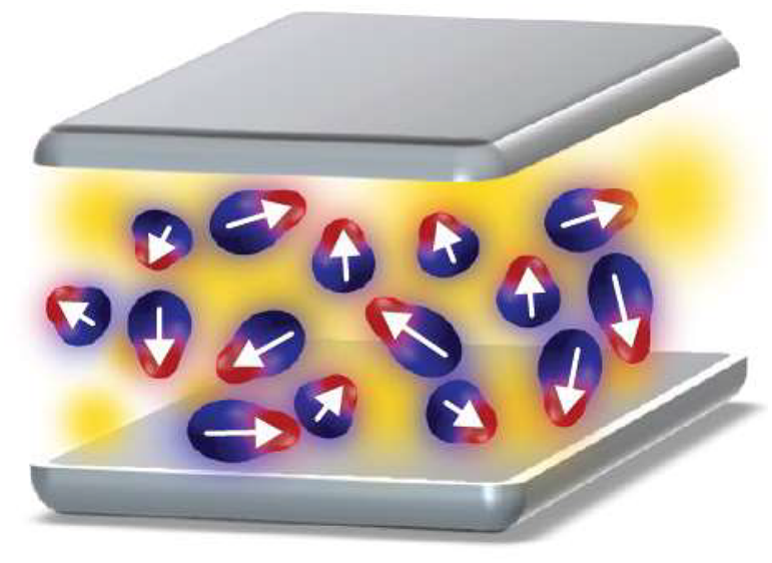

We demonstrate that collective vibrational strong coupling of molecules in thermal equilibrium can give rise to significant local electronic polarizations in the thermodynamic limit. We do so by first showing that the full nonrelativistic Pauli–Fierz problem of an ensemble of strongly coupled molecules in the dilute-gas limit reduces in the cavity Born–Oppenheimer approximation to a cavity–Hartree equation for the electronic structure. Consequently, each individual molecule experiences a self-consistent coupling to the dipoles of all other molecules, which amount to non-negligible values in the thermodynamic limit (large ensembles). Thus, collective vibrational strong coupling can alter individual molecules strongly for localized ”hotspots” within the ensemble. Moreover, the discovered cavity-induced polarization pattern possesses a zero net polarization, which resembles a continuous form of a spin glass (or better *polarization glass*). Our findings suggest that the thorough understanding of polaritonic chemistry, requires a self-consistent treatment of dressed electronic structure, which can give rise to numerous, so far overlooked, physical mechanisms.

Polaritonic chemistry and materials science is a rapidly growing research field evidenced by a large number of recent review articles.^1−11^ The strong coupling of matter and light within optical cavities offers a novel way not only to alter and design matter properties, but also to shape the (quantum) properties of light in various ways. For example, magnetic^12^ or metal-to-insulator^13^ phase transitions can be altered. Furthermore, cavities can also cause the breakdown of topological protection as reported for the integer quantum Hall effect.^14^ In chemistry, the electronic strong coupling, the quantum yield of emissions^15^ or intersystem crossings^16^ can be modified and photochemical reactions can be influenced.^17−22^ For vibrational strong coupling even ground-state (thermally driven) chemical reactions can be affected^23−26^ However, despite a plethora of suggested applications and observed novel effects, we still lack a fundamental understanding of all the relevant underlying microscopic/macroscopic physical mechanisms, specifically in the context of vibrational strong coupling effects.^6,27,28^ One of the main reasons for this deficiency is the complexity of the full description, which a priori requires a holistic approach combining the expertise from different fields of physics and chemistry such as quantum optics, electronic structure theory, (quantum) statistical mechanics, quantum electrodynamics, and molecular and solid state physics.^11^ Besides questions concerning the observed resonance conditions,^29−32^ currently one of the most pressing, unresolved issues in the field is how individual molecules can experience cavity-induced modifications under collective strong coupling.^6,11,27,28^ Theoretical attempts to determine how the coupling of the cavity to the ensemble of molecules can modify the chemistry of individual molecules in the thermodynamic limit have so far only been able to describe certain aspects.^33−36^ While there have been theoretical suggestions that collective strong coupling can lead to local changes once impurities or (thermally induced) disorder is introduced in an ensemble,^37,38^ the existence and nature of such effects for a large ensemble of molecules has remained elusive. In this letter we close this important gap by demonstrating numerically that the cavity can indeed induce local polarization effects akin to those observed for small molecular ensembles^38^ for collective coupling in the thermodynamic limit, when treating the many-molecule problem self-consistently within the cavity Born–Oppenheimer approximation of the full Pauli–Fierz theory.

We consider a dilute gas-phase ensemble of *N* molecules coupled to a photonic environment with confined modes α. Each molecule consists of *N*_e_ electrons and *N*_n_ nuclei/ions such that in the long-wavelength limit the Pauli–Fierz Hamiltonian becomes^39−41^
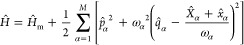
1with *Ĥ*_m_ the usual cavity-free/bare matter Hamiltonian consisting of *N* molecules. The coupled polarization operators are defined as *X̂*_α_ ≔ ∑_*n*=1_^*N*^ ∑_*i*_*n*_=1_^*N*_*n*_^*Z*_*i*_*n*__**λ**_α_·***R̂***_*i*_*n*__ and *x̂*_α_ ≔ −∑_*n*=1_^*N*^ ∑_*i*_*n*_=1_^*N*_e_^**λ**_α_·***r̂***_*i*_*n*__, where *Z*_*i*_*n*__ is the nuclear charge and ***R̂***_*i*_*n*__ is the coordinate of the *i*th nucleus/ion of the *n*th molecule and accordingly for the electrons **r̂**_i_*n*__. The coupling strength and polarization to the canonical displacement field operators *q̂*_α_ and *p̂*_α_ with mode frequency ω_α_ are defined by **λ**_α_ and can be obtained from, e.g., macroscopic quantum electrodynamics.^42,43^

In the next step we perform the cavity Born–Oppenheimer approximation (cBOA);^44−46^ i.e., we treat the electrons of the ensemble as a conditional many-body wave function of all of the nuclear degrees of freedom **R** and all of the displacement field coordinates *q*.^12^ We subsequently assume the dilute-gas limit and thus the overlaps of *local* many-electron ground-state wave functions |χ_*n*_⟩, of different molecules is considered negligible, and thus a mean-field ansatz for the *ensemble* electronic wave function
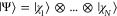
2becomes accurate (see the Supporting Information (SI)). We note that this ansatz leads to the same set of equations as a Slater determinant of *all* electrons, where we assume that the individual electronic wave functions of different molecules do not overlap. This leads to a set of coupled equations, where the *local* electronic structure of the *n*th molecule depends self-consistently on all the *N* – 1 other molecules. Disregarding bare molecular interaction in the dilute limit, we then have to find the lowest electronic energy state for the following cavity-Hartree equations
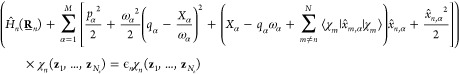
3for all *N* molecules simultaneously, i.e., by a self-consistent solution. eq 3 can be solved analytically for an ensemble of *N* simple harmonic (model) molecules, which is discussed in ref (47). The bare matter Hamiltonian of a single molecule is defined as *Ĥ*_*n*_ from *Ĥ*_m_(**R**) = ∑_n=1_^*N*^*Ĥ*_*n*_(**R**_*n*_) within the dilute-limit approximation and *x̂*_*n*,α_ = −∑_*i*_*n*_=1_^*N*_*e*_^**λ**_α_·***r̂***_*i*_*n*__ is the electronic polarization operator of the *n*th molecule and **z** = **r** σ the space–spin variable of an individual electron. Consequently, the cavity induces an intermolecular dipole–dipole energy term of the electronic structure
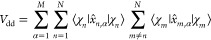
4that scales with *N* (*N* – 1) over the entire ensemble size. This macroscopic scaling is crucial for molecular ensembles, since it counteracts the usual 1/√*N* scaling law of the coupling terms **λ**_α_ for a fixed Rabi splitting, as we show subsequently. Notice that the nuclear dipole–dipole interaction term is separated off in the cBOA partitioning of the full quantum problem given in eq 1 and treated classically in our case (see eq S2 in the Supporting Information). Indeed, the intermolecular dipole–dipole interaction is physically straightforward to understand. Because we work in the length gauge, the electric field operator is^48^
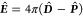
5The effect of the macroscopic polarization ***P̂*** = −∑_α=1_^*M*^**λ**_α_(*X̂*_α_+*x̂*_α_)/(4π) on the microscopic constituents is captured by the dipole self-interaction and scales as **λ**_α_^2^. That these self-interaction terms are important to properly describe the optical response of a material system has been pointed out earlier.^49^ In addition, it is also established that disregarding this term (as often done in model calculations) and only keeping the cavity-mediated displacement field ***D̂*** = ∑_α=1_^*M*^**λ**_α_ω_α_*q̂*_α_/(4π) introduces severe theoretical inconsistencies for ab initio simulations.^48,50^ In the following, we will investigate the chemical relevance of treating ***P̂*** self-consistently on a macroscopic and a microscopic scale.

From the self-consistent solution of eq 3 for all *N* molecules, we obtain the classical forces for the nuclei/ions and the displacement-field coordinates. In more detail, we can perform an ab initio molecular dynamics simulation on the polarization-dressed ground-state potential energy surface. To account for classical canonical equilibrium conditions at temperature *T*, which are relevant for many ground-state chemical processes, the classical Langevin equations of motion are propagated; i.e.,^6,51^
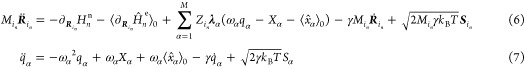
These equations implicitly assume that the Hellmann–Feynman theorem applies, i.e., that the cavity Hartree equations are not only globally but also locally solved using a variational method. The bare matter Hamiltonian *Ĥ*_*n*_ is separated into a classical part, describing nuclear interactions *H*_*n*_^n^, and the quantized electronic part *Ĥ*_*n*_^e^ that parametrically depends on the nuclear positions. Furthermore, we have introduced nuclear masses *M*_*i*_*n*__, friction constant γ, and component-wise δ-correlated Gaussian noise terms, i.e., ⟨*S*(*t*)⟩ = 0, ⟨*S*(*t*) *S*(*t*′)⟩ = δ(*t* – *t*′). Each degree of freedom possesses its individual independent stochastic noise term indexed by *i*_*n*_ and α, respectively. We note here that treating the displacement coordinates classically with thermal noise means that we consider photonic excitations due to free charges to be in a classical thermal state.^48^

In the first step, the collective Rabi splitting is calculated for a model system consisting of *N* = 900 randomly oriented and slowly rotating Shin–Metiu molecules^52^ strongly coupled to a single cavity mode ω_α_ at *T* = 158 K, yielding a clear lower and upper polaritonic resonance, as depicted by the dotted line in Figure 1a (see the SI for further details). Notice, the differently aligned molecules in the dilute limit can also be reinterpreted as aligned molecules coupled to a spatially modulated displacement field, which makes our results more generally applicable. Taking into account the self-consistent treatment of the cavity-induced molecular polarization ***P̂*** leads to a detuning of the cavity toward lower frequencies, which manifests itself in the asymmetric splitting with respect to the bare cavity mode ω_α_, indicated by the black vertical line. The red shift of the cavity frequency can directly be related to the refractive indesx of the ensemble within the harmonic approximation.^47,53^ Simulations of a few less polarizable hydrogen fluoride (HF) dimers^54,55^ show the same tendency as demonstrated in Figure 1b and Figure S3, but of significantly smaller magnitude. Qualitatively similar results can also be found for perfectly aligned Shin–Metiu molecules (see Figure S1 in the SI). The observed cavity induced detuning resembles the dipole–dipole interaction induced Lorentz red shift within dense atomic ensembles (in the absence of a cavity), which depends strongly on the microscopic polarizability of the media, similar to our result.^56,57^ Notice that when tuning the cavity to much lower frequencies (e.g., ro-vibrational regime), the presence (back-action) of permanent molecular dipoles is expected to significantly contribute to the red shift alongside the molecular polarizability. This dynamic reorientation contribution is neglected in our simulations. The observed collectively induced red shift of a filled cavity with respect to a bare one has also been seen in experiments^58^ and may in principle be simply approximated by a suitably chosen refractive index of the ensemble,^6^ with perfect agreement for harmonic molecules as discussed in ref (47). We also note that here we get the red shift directly from the simulation, where we calculate the self-consistent polarization of the ensemble of molecules. That is, we calculate implicitly the ensemble polarizability and its back-action on the cavity mode. In the case of free-space modes, this is the standard way to determine the refractive index of a material.^49,59,60^ Now the question arises, Can the accurate self-consistent and microscopic treatment of the polarization additionally induce local field effects that cannot be disregarded in the thermodynamic limit (*N* ≫ 1) and that are not captured by a simple refractive index picture? Earlier evidence for collective electronic strong coupling for a few molecules indeed indicates that there might be such an effect,^38^ yet the existence of similar local polarization effects for a thermal ensemble under vibrational strong coupling in the large-*N* limit remained unclear. As the first local observable, we analyze the local molecular dipole vibrations for individual Shin–Metiu molecules, which reveals a (locally) populated lower polariton (solid line in Figure 1a) and a strongly populated dark state at ω = ω_α_. A local upper polariton could not be identified, i.e., may be too weakly populated to overcome the thermal broadening for the given system. Simulations show that the usual √*N*-collective Rabi split scaling law of the Tavis–Cumming model remains preserved collectively as well as locally, when including local polarization effects self-consistently (see Figure S1 of the Supporting Information). As the second local observable, we propagate the system self-consistently and measure at every time step, i.e., for every realized classical configuration (*R*, *q*_α_), the difference between the exact solution of eq 3 and the electronic bare matter problem by monitoring Δ*r*_*n*_(*t*) = ⟨*r̂*_*n*_⟩_λ_ – ⟨*r̂*_*n*_⟩_λ=0_ in the electronic ground state. This allows measurement of cavity-induced local polarization effects in thermal equilibrium, since the full electronic problem reduces to the bare local matter problem in the thermodynamic limit if only the displacement field is considered instead. Our simulation results in Figure 2 revealing a nonvanishing cavity-induced local ensemble polarization, i.e., ⟨|Δ*r*_*n*_|⟩ ≠ 0, that persists even in the large-*N* limit. At the same time, the total electronic polarization of the ensemble remains zero; i.e., ⟨Δ*r*_*n*_⟩ = 0, as expected from the symmetry of eq 1. Consequently, our numerical results show that the chemical properties of individual molecules can be locally modified by collective strong vibrational coupling to the cavity. Fundamentally speaking, our observation of a continuous distribution of cavity-induced molecular polarizations with zero net polarization resembles a continuous form of a spin-glass^61^ (or better *polarization-glass*). The continuous distribution automatically implies the existence of *hotspots* within the molecular ensemble, where the collective coupling can strongly polarize single molecules and thus significantly alter their chemical properties. However, the average cavity-induced polarization remains rather small, which seems in agreement with recent NMR experiments.^62^ Notice this collectively induced local mechanism occurs without external driving; i.e., the sole presence of a thermal bath is sufficient. The detailed study of the physical properties of the hotspots and the polarization glass (e.g., thermodynamics, implied time scales, distribution, and frustration effects) will be left for future work, since, in analogy with spin-glasses,^61^ these are most likely highly nontrivial, as well as strongly interconnected theoretical aspects, which will require considerable efforts. Analogous results hold also for perfectly aligned molecules, as shown in Figure S2 of the Supporting Information. Physically, the appearance of local strong-coupling effects can be understood by interpreting the local polarization in a dipole picture, as previously done for electronic strong coupling of a few nitrogen dimers.^38^ While the total (macroscopic) polarization is zero, nontrivial local dipole modifications are possible for heterogeneous systems that can still cancel each other, i.e., as seen from the sum *m* ≠ *n* in eq 3. This local polarization induces a mirror dipole in the rest of the ensemble. At this point, we highlight the relevance of (random) disorder in the ensemble (temperature and/or different molecular orientations, vibrational states, and polarizabilities), which enables a heterogeneous structure of modified local polarizations that can cancel collectively in analogy to a spin-glass.^63^ For atoms, which do not have a static dipole moment, no local effect is expected, as can be confirmed by simulating a small ensemble of up to five neon atoms (see Figure S4 in the Supporting Information). In other words, having spherically symmetric systems without (different) internal nuclear degrees of freedom, all local dipole contributions will be equivalent and thus the local polarization needs to be zero, in order to have a zero macroscopic polarization. Furthermore, the simple harmonic model considerations in ref (47) demonstrate another important ingredient for the formation of polarization glass, which is the presence of a complex electronic structure; i.e., anharmonic electron interactions (e.g., Coulomb) are mandatory. Overall, our results do not contradict well-established knowledge from quantum-optical models for *atomic* systems. However, they show that, for molecular ensembles, the formalism becomes more involved and the self-consistent (!) treatment of the local polarization may become decisive in capturing all relevant aspects of polaritonic chemistry. We also note that the free-space mode structure of the electromagnetic field, which is homogeneous and isotropic, is not able to test for disorder in the same way as cavity modes do by having preferred polarization directions and frequencies. This breaking of symmetry explains why similar effects are not expected for coupling to free-space modes.

**Figure 1:**
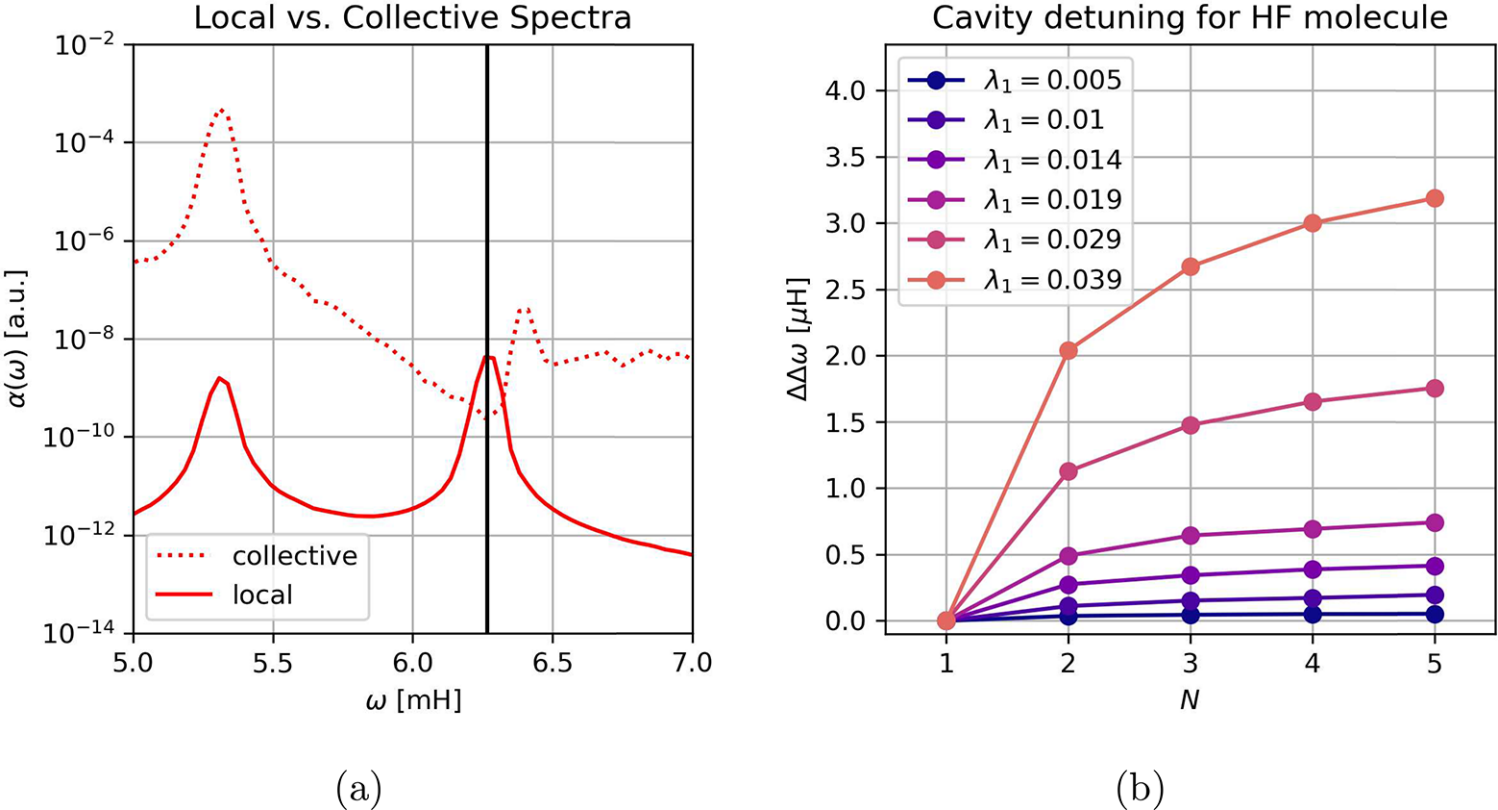
(a) Vibrational absorption spectra α(ω) for 900 randomly oriented Shin–Metiu molecules under collective vibrational strong coupling in canonical equilibrium at *k*_B_*T* = 0.5 mH. The cavity is tuned to the first vibrational mode of the uncoupled molecule at ω_α_ = 6.27 mH (black vertical line) with λ_α_ = 0.0085. The collective Rabi splitting (dotted line) is calculated from the fluctuations of the total ensemble dipole moment (see SI) and shows an asymmetric splitting (red-shifted cavity). In addition, local molecular vibrations (bold line) are monitored in a similar way (see SI), which reveals a significant fraction of individual molecules that are locally strongly coupled, i.e. that vibrate at the frequency of the lower polariton. Furthermore, the local spectrum also indicates that the dark states at ω = ω_α_ are strongly populated. In contrast, no local populations of the upper polaritonic states could be detected at the given temperature. (b) Relative red-shifted cavity frequencies ΔΔω = |Δω(*N*) – Δω(1)| with respect to the single molecule shift Δω(1) for a few perfectly parallelly aligned HF molecules. The collective Rabi splitting was kept constant with respect to *N* for each chosen λ_1_ by rescaling  throughout the computations. The cavity is tuned to the first vibrational mode of the uncoupled HF at ω_α_ = 20.35 mH (see SI for further details). Notice the detuning is about 2 orders of magnitude smaller for the HF molecule than for the Shin–Metiu molecule. However, the results agree qualitatively, since they suggest a similar finite collective detuning in the large *N* limit. The overall very small shift is a consequence of the very low polarizability 0.8 Å^3^ of the HF molecules.^76^

**Figure 2:**
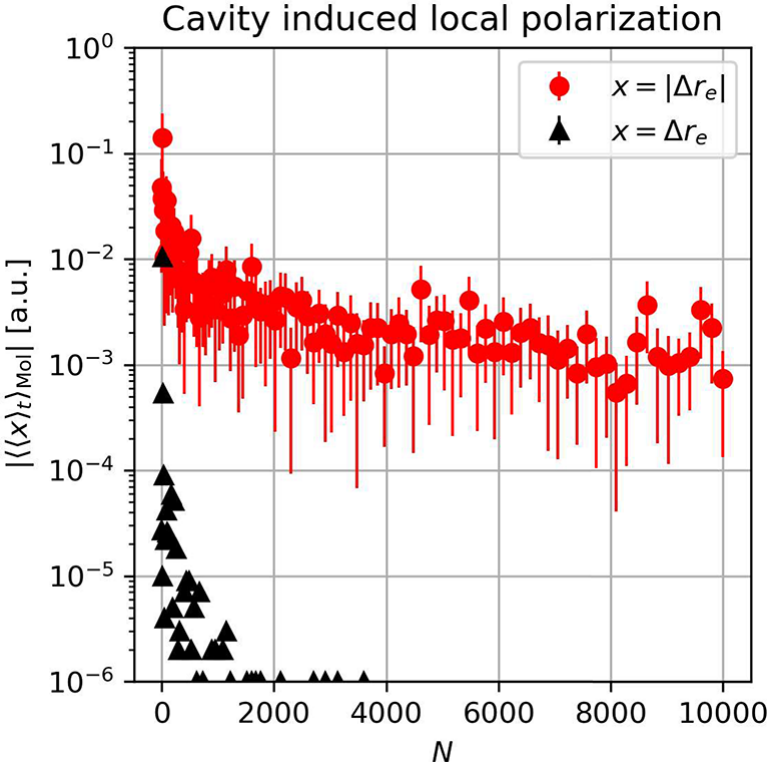
Statistical evaluation of cavity-induced local electronic changes Δ*r*_*n*_(*t*) = ⟨*r̂*_*n*_⟩_λ_ – ⟨*r̂*_*n*_⟩_λ=0_ of the *n*th molecule with respect to the bare Shin–Metiu molecule in canonical equilibrium at *k*_B_*T* = 0.5 mH for randomly oriented molecules (see Supporting Information for details). The Rabi splitting was kept constant when increasing the number of molecules by choosing a rescaled . By monitoring |Δ*r*_*n*_| (red dots), we observe a nonzero saturation of the cavity-induced local polarizations in the large-*N* limit, where the standard deviations with respect to different molecules are indicated by vertical red lines. At the same time, the total polarization of the ensemble, which is related to Δ*r*_*n*_ (black triangles), quickly approaches zero, since the cavity cannot induce a nonzero polarization of the ensemble. Consequently, our simulations suggest that cavity-induced local strong coupling effects persist in the thermodynamic limit (*N* ≫1) of a molecular ensemble under collective vibrational strong coupling. In other words, the self-consistent treatment of eq 3 is decisive to describe ground-state polaritonic chemistry accurately for realistic molecular ensembles.

To conclude, we have reformulated the computationally inaccessible many-molecule Pauli–Fierz problem of polaritonic chemistry in terms of an efficient cavity–Hartree many-molecule problem, within the dilute-gas limit and the cBOA. Simulating the corresponding Langevin equations of motion under vibrational strong coupling in thermal equilibrium reveals that solving the cavity–Hartree equations self-consistently, and thus including dipole–dipole interactions between molecules, can be decisive for capturing all relevant aspects of polaritonic chemistry. The reason is that nontrivial local (on the individual-molecule level) polarization distributions can arise with zero net polarization, which can persist in the thermodynamic limit and thus may be regarded as a *polarization-glass* phase. The continuous distribution implies the existence of molecular *hotspots*, where chemistry is locally altered significantly by the cavity. Furthermore, our self-consistent accounting for ensemble polarization effects leads to a detuning of the cavity toward lower frequencies, which is in line with experimental evidence^58^ and shows that the dipole self-interaction term is a necessary ingredient to capture the basic effect of a changed refractive index.

The present result may have far-reaching consequences for the theoretical description of polaritonic chemistry and materials science, since they provide a so far overlooked, yet simple and intuitive, physical mechanism that can induce local molecular changes in the thermodynamic limit. This local mechanism may be the missing piece to settle current discrepancies between existing simplified models for a macroscopic ensemble of molecules and experiments. Furthermore, our cavity–Hartree equations are well suited to be included in existing computational methods,^40,45,64−69^ which will enable the efficient exploration of the large chemical space with a multitude of observables. Particularly, large ensemble sizes under self-consistent vibrational strong coupling should become accessible by established ab initio molecular dynamics codes^65−67,70^ and potentially with the help of self-consistent embedding schemes.^71^ Last, but not least, the existence of a macroscopically induced microscopic polarization mechanism opens many interesting fundamental physical questions. For instance, can we efficiently control microscopic (quantum) properties of individual molecules via a thermal macroscopic field or are the experimentally observed modifications of chemical reactions purely due to change in the statistics? Can computationally efficient, single molecular polaritonic rate theories^72−74^ be applied on the molecular *hotspots* and are there polarization-glass-induced thermodynamic changes that need to be included as well? On the more theoretical side, can our results be generalized to the liquid or even solid phase under collective strong coupling conditions? What are the physical properties of the suggested cavity-induced *polarization-glass* phase and its relation to a spin glass? Can thus the thermodynamics of molecules under VSC be affected akin to frustration in a spin-glass (e.g., breakdown of fluctuation–dissipation theorem with excess of thermal fluctuations)?^61,75^ All of these aspects open many interesting questions that lie at the boundaries between physics and chemistry and need the combination of various different viewpoints and methods.^2^

## Supporting Information Available

Theoretical and computational details including the choices of simulation parameters; additional simulation results for an aligned Shin–Metiu setup as well as for the HF molecules under VSC (PDF)
Transparent Peer Review report available (PDF)
