# Supplementary material for: Unraveling a Cavity-Induced Molecular Polarization Mechanism from Collective Vibrational Strong Coupling
Source: J Phys Chem Lett. 2024 May 8;15(19):5208–14. doi: 10.1021/acs.jpclett.4c00913 (PMC11103705; doi:10.1021/acs.jpclett.4c00913)
Supplement: Supplementary file 1 — jz4c00913_si_001.pdf [file jz4c00913_si_001.pdf]

# SUPPORTING INFORMATION: Unraveling a Cavity-Induced Molecular Polarization Mechanism from Collective Vibrational Strong Coupling

Dominik Sidler,[1,2,3] Thomas Schnappinger,[4] Anatoly Obzhirov,[2,3] Michael Ruggenthaler,[2,3] Markus Kowalewski,[4] and Angel Rubio[2,3,5,6]

[1)] *Laboratory for Materials Simulations, Paul Scherrer Institute, 5232 Villigen PSI, Switzerland*

[2)] *Max Planck Institute for the Structure and Dynamics of Matter and Center for Free-Electron Laser Science, Luruper Chaussee 149, 22761 Hamburg, Germany*

[3)] *The Hamburg Center for Ultrafast Imaging, Luruper Chaussee 149, 22761 Hamburg, Germany*

[4)] *Department of Physics, Stockholm University, AlbaNova University Center, SE-106 91 Stockholm, Sweden*

[5)] *Center for Computational Quantum Physics, Flatiron Institute, 162 5th Avenue, New York, NY 10010, USA*

[6)] *Nano-Bio Spectroscopy Group, University of the Basque Country (UPV/EHU), 20018 San Sebastián, Spain*

(*Electronic mail: angel.rubio@mpsd.mpg.de)

(*Electronic mail: markus.kowalewski@fysik.su.se)

(*Electronic mail: michael.ruggenthaler@mpsd.mpg.de)

(*Electronic mail: anatoly.obzhirov@mpsd.mpg.de)

(*Electronic mail: thomas.schnappinger@fysik.su.se)

(*Electronic mail: dsidler@mpsd.mpg.de)

(Dated: 7 May 2024)

## I. CAVITY HARTREE EQUATIONS

In order to derive the cavity Hartree Eq. (3) in the letter, we start from the fully quantize Pauli-Fierz Hamiltonian given in Eq. (1) and apply a Born-Huang expansion to the total wave function following Ref. 1. In more detail, we separate the electronic degrees of freedom from the nuclear-photonic degrees of freedom yielding $\Psi_i = \sum_{j=1}^{\infty} \gamma_{ij}(\mathbf{R},q)\psi_j(\mathbf{r},\mathbf{R},q)$ that solves $\hat{H}\Psi_i = E_i\Psi_i$. Afterwards, we employ the cavity Born-Oppenheimer approximation in its simplest form, which assumes a classical treatment of the nuclear and photonic degrees of freedom.[1] This approximation accurately describes cavity mediated ground-state chemistry, i.e. when the cavity is tuned on the vibrational degrees of freedom and the electronic excitations are sufficiently separated energetically such that non-adiabatic couplings (e.g. conical intersections) are negligible.[1–5] Consequently, the system separates into an electronic subsystem $\hat{H}^{\mathrm{e}}$, which parametrically depends on the modes $\underline{q}$ and the nuclear position vector $\underline{\boldsymbol{R}}$, and into a nuclear-photonic part $\hat{H}^{\mathrm{npt}}$. Following the notation of the letter, the resulting electronic Hamiltonian operator becomes

$$\hat{H}^{\mathrm{e}} := \hat{H}^{\mathrm{e}}_{\mathrm{m}} + \sum_{\alpha=1}^{M}\left(\frac{1}{2}\hat{x}_{\alpha}^{2} + \hat{x}_{\alpha}X_{\alpha} - \omega_{\alpha}\hat{x}_{\alpha}q_{\alpha}\right), \tag{S1}$$

which depends parametrically on $\mathbf{R}, q_{\alpha}$. The corresponding groundstate Hamiltonian of the coupled nuclear-photon degrees of freedom is given by,

$$H^{\mathrm{npt}} := H^{\mathrm{n}}_{\mathrm{m}} + \sum_{\alpha=1}^{M}\left(\frac{p_{\alpha}^{2}}{2} + \frac{\omega_{\alpha}^{2}}{2}\left(q_{\alpha} - \frac{X_{\alpha}}{\omega_{\alpha}}\right)^{2} + \langle\psi_0|\hat{H}_e(\underline{\mathbf{R}},\underline{q})|\psi_0\rangle\right). \tag{S2}$$

In a next step, we demonstrate that the groundstate many-electron eigenvalue problem imposed by $\hat{H}^{\mathrm{e}}$ can be solved exactly in the dilute limit by a simple Hartree product ansatz. To show this, we start from a Hartree-Fock mean-field Ansatz[6] given in Eq. (2) of the letter to solve for $\varepsilon_0 = \min E_{HF} = \min\langle\psi_{HF}|\hat{H}^{\mathrm{e}}|\psi_{HF}\rangle$. The resulting Hartree-Fock energy can then simply be written as,

$$\begin{aligned} E_{HF} &= \sum_{n=1}^{N}\langle\chi_n|\hat{H}^{\mathrm{e}}_{n} + \sum_{\alpha=1}^{M}\left[\frac{1}{2}\hat{x}_{n,\alpha}^{2} + \hat{x}_{n,\alpha}X_{\alpha} - \omega_{\alpha}\hat{x}_{n,\alpha}q_{\alpha}\right]|\chi_n\rangle \\ &+\frac{1}{2}\sum_{n,m}^{N}\sum_{\alpha=1}^{M}\langle\chi_n|\hat{x}_{n,\alpha}|\chi_n\rangle\langle\chi_m|\hat{x}_{m,\alpha}|\chi_m\rangle - \cancel{\frac{1}{2}\sum_{n,m}^{N}\sum_{\alpha=1}^{M}\langle\chi_n|\hat{x}_{n,\alpha}|\chi_m\rangle\langle\chi_m|\hat{x}_{m,\alpha}|\chi_n\rangle}, \end{aligned} \tag{S3}$$

where the cavity-induced electron-electron exchange cancel in the last terms, except for $n = m$, due to the dilute limit assumption, i.e, the electronic structure of different molecules must not overlap. Notice that for non-overlapping electronic structures the inter-molecular exchange and correlation energies are zero, and if the molecules are far apart also the Coulomb Hartree energy

goes to zero. Therefore we have $\hat{H}^{\mathrm{e}}_{\mathrm{m}}$ simplifies to $\hat{H}^{\mathrm{e}}_{\mathrm{m}} = \sum_{n=1}^{N} \hat{H}^{\mathrm{e}}_{n}$ in Eq. (S3). Consequently, our mean-field ansatz becomes exact in the dilute limit. However, the exact solution of the local bare matter problem, i.e., finding accurate eigenfunctions of $\hat{H}^{\mathrm{e}}_{n}$, may itself be a highly non-trivial problem that requires to consider the intra-molecular correlations with computationally expensive post Hartree-Fock methods. From Eq. (S3) we find the resulting Hartree equations for the $n$-th orbital upon variation of the orbitals $\chi$ as presented in Eq. (3) of the letter. Orthogonality conditions $\langle \chi_n | \chi_m \rangle = \delta_{nm}$ are automatically obeyed, i.e., our orbitals are automatically canonical, since we assume non overlapping orbitals. Eventually, we are ready to perform an ab-initio molecular dynamics simulation on the exactly dressed ground-state potential energy surface in classical canonical equilibrium by time-propagation of the standard Langevin equations of motion as given in Eqs. (6) and (7) of the letter. For the force calculation, we use that the Hellmann-Feynman theorem applies for the variational cavity Hartree eigenvalue problem, provided that the local eigenvalue problem of the $n$-th molcecule can be solved with a variational method (as we do for the Shin-Metiu molecule by exact diagonalization below).

## II. CAVITY AB-INITIO MOLECULAR DYNAMICS FOR RANDOM ORIENTED SHIN-METIU MOLECULES

To mimic the impact of rotational molecular disorder on collective vibrational strong couplig, we simulated rotational molecular motion at finite temperature by an overdamped rotational Langevin equation, i.b. by rotational Brownian motion. This can efficiently be done for 1D Shin-Metiu molecules by introducing a time-dependent coupling constant

$$\lambda_{\alpha,n}(t) = \boldsymbol{\lambda}_{\alpha} \cdot \mathbf{n}_{n}(t), \tag{S4}$$

for each individual molecule $n$. We have assumed a cavity polarization along the $z$-axis, i.e., $\boldsymbol{\lambda}_{\alpha} = \lambda_{\alpha}\mathbf{e}_{z}$ and that the 1D molecule should be aligned in 3D along the $\mathbf{n}_{n}(t)$-direction. Notice that there is no feedback from the molecular and/or cavity state onto the normalized orientation $\mathbf{n}_{n}(t)$. In other words, we assume in the following that the rotational motion is solely determined by the rotational Debey relaxation time $\tau_R = 2\pi/\omega_R$, which we will choose considerably smaller than the timescale of the nuclear vibrations (off-resonant with respect to the tuning of the cavity). However, the slowly changing random molecular orientation, will certainly influence the collective strong coupling effects between the molecules and the cavity. To mimic random rotational motion

by $\mathbf{n}_n(t)$ the rotational Langevin equation in the overdamped limit can be used, i.e., as,[7,8]

$$\frac{d\boldsymbol{n}_n(t)}{dt} = \sqrt{\tau_R}\boldsymbol{S}_n(t) \times \boldsymbol{n}_n(t) \tag{S5}$$

with Debye relaxation time $\tau_R = 2k_BT/\eta_R$ and unbiased, delta-correlated Gaussian noise sources $\boldsymbol{S}(t)$. The rotational friction coefficient is labeled by $\eta_R$. This first order stochastic PDE is propagated numerically using forward Euler method in a similar spirit to Ref. 9, for which we yield

$$\boldsymbol{n}_{n,t+\Delta t} = \frac{\boldsymbol{n}_{n,t}}{|\boldsymbol{n}_{n,t}|} + \sqrt{\Delta t\,\tau_R}\boldsymbol{S}_{n,t} \times \frac{\boldsymbol{n}_{n,t}}{|\boldsymbol{n}_{n,t}|}. \tag{S6}$$

and exact normalisation is imposed at every time-step. Eqs. (S4) and (S6) then allow the simple simulation of vibrational strong coupling for a randomly oriented molecular ensemble within our self-consistent cBOA approach.

## III. SIMULATION SETUP

### A. Shin-Metiu molecular dynamics simulation

The Hamiltonian operator $\hat{H}_{\mathrm{SM}}$ of the $n$-th Shin-Metiu molecule is given by,[10,11]

$$\begin{aligned}\hat{H}_n &= \frac{\hat{P}^2}{2M} + \frac{\hat{p}^2}{2} + \frac{1}{|L/2-\hat{R}|} + \frac{1}{|L/2+\hat{R}|} \\ &- \frac{\mathrm{erf}(|\hat{R}-\hat{r}|/R_f)}{|\hat{R}-\hat{r}|} - \frac{\mathrm{erf}(|\hat{r}-L/2|/R_r)}{|\hat{r}-L/2|} - \frac{\mathrm{erf}(|\hat{r}+L/2|/R_l)}{|\hat{r}+L/2|}.\end{aligned} \tag{S7}$$

We have used atomic units throughout our calculations with $R_f = R_l = R_r = 1.511$ and $L = 9.45$. We chose the proton mass $M = 1836$ for the moving nuclei with positive unit charge and $m = 1$ for the electron with negative unit charge. For the 1D electron, we chose a converged grid basis set representation with 41 equidistant grid points and a grid spacing of 0.8. Notice that having a large enough basis set for the electrons is pivotal to resolve local polarization effects.[12] The cavity Hartree equations were minimized self-consistently until converging the total electronic ensemble energy up to $\Delta E < 1 \times 10^{-7}$. The classical Langevin equations of motion were propagated numerically using the scheme of Ref. 13 with a time step $\delta t = 50$. Trajectories were simulated over 2000 time-steps to evaluate the local polarization effects and for 50000 time-steps for the spectra calculations. Nuclei were initialized randomly distributed in the vicinity of the ground-state. Thermostating parameters were set to $k_BT = 0.5 \times 10^{-3}$ with low friction coefficient $\gamma = 0.3 \times 10^{-5}$ (underdamped regime). In case of randomly oriented molecules, the Debye relaxation time was

set to $\tau_R = 0.5 \times 10^{-5}$. Notice that the parametrization of the Shin-Metiu molecule and the temperature was chosen such that non-adiabatic coupling effects should not play a role, i.e., the ground-state cBOA approximation is valid. Furthermore, the temperature was chosen small enough that the thermal broadening does not interfere with our spectral interpretation. In addition, no chemical reaction occurs at such low temperatures on the chosen time-scale (no proton transfer between the two energy minima of the Shin-Metiu molecule). The impact of local polarization effects on chemical reaction rates will be the focus of future work instead.

The vibrational absorption spectra was calculated using the power spectra method in Ref. 14 with a Blackman filter window[15] averaged over 33 overlapping trajectory windows containing 4096 time steps, each of them shifted by $1/3$ of the window size. While for the global absorption spectrum the total dipole (electronic + nuclear contribution) were post-processed accordingly (doted lines), we did the same for each individual molecular dipole in case of the local spectra calculation instead. Afterwards, the summation over all local spectra was taken yielding the bold lines in the spectral figures.

Supplementary simulation results for aligned Shin-Metiu molecules (spectra, Rabi-split scaling and local polarizations) are shown in Figs. S1 and S2.

## B. Ensembles of HF molecules and Ne atoms

The cBOA Hartree-Fock method[6] corresponding analytic nuclear gradients[16] were implemented in the Psi4NumPy environment[17], which is an extension of the PSI4[18] electronic structure package. All calculations were performed using the aug-cc-pVDZ basis set[19] and the geometry of the isolated single HF molecule is optimized at the Hartree-Fock level of theory. Note that we have not reoptimized the geometries of the molecular systems in the cavity; as such, our calculations do not account for any geometric relaxation effects stemming from the presence of the cavity. In all cavity Hartree-Fock calculations performed in this work, we consider a single-mode and non-lossy cavity. The fundamental cavity frequency $\omega_\alpha$ is tuned to the first vibrational mode of the uncoupled HF at 20.35 [mH].

For the molecular ensembles studied, the optimized structure of a single HF molecule is replicated $N$ times. All these replicas are separated by 800 Å and placed inside a cavity. All individual molecular dipole moments are aligned with the cavity polarization axes, and the zero transversal electric field condition is satisfied for the whole ensemble. The same distance of 800 Å is used

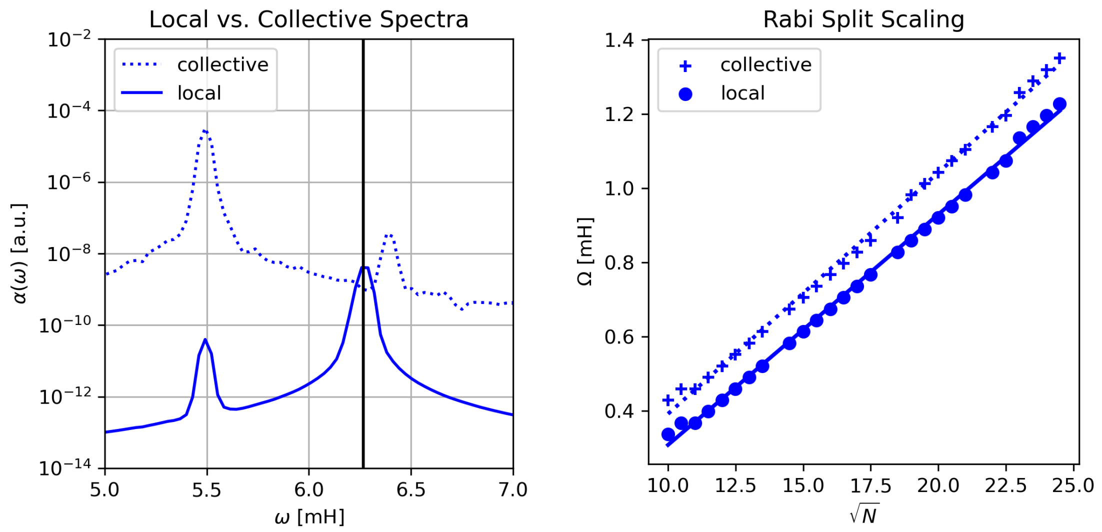


(a) (b)

FIG. S1: (a) Vibrational absorption spectra $\alpha(\omega)$ for 900 aligned Shin-Metiu molecules under collective vibrational strong coupling similar to the random oriented molecules shown in the letter. The cavity is again tuned on the first vibrational mode of the uncoupled molecule at $\omega_\alpha = 6.27$ [mH] (black vertical line). However, the coupling is reduce to $\lambda_\alpha = 0.00425$ in order to achieve a Rabi splitting of similar magnitude. We find qualitative identical results for the aligned molecules as for the random oriented setup, i.e. a de-tuning of the cavity to lower frequencies in combination with a locally populated lower polaritonic state.
(b) The collective $\sqrt{N}$-scaling law of the Rabi splitting remains preserved, when solving the self-consistently the coupled electronic problem for the molecules under vibrational strong coupling. Similarly, the observed local splitting follows the same $\sqrt{N}$-scaling law, where we defined the splitting between (local) lower polariton and the dark states located at $\omega_\alpha$. This suggests that local strong coupling can be increased by collectively enhancement of the coupling. Small discrete patterns in the data emerge from finite spectral resolution of the discrete Fourier transformation applied to dipole trajectories of finite length.

for the small ensembles of neon atoms. The vibrational spectra for the cavity-coupled ensembles are calculated in the harmonic approximation using numerical second derivatives of the cavity Hartree-Fock energy[16]. All calculations were performed in a reproducible environment using the Nix package manager (nixpkgs, 22.11, commit 594ef126) in combination with NixOS-QChem (commit f5dad404).[20]

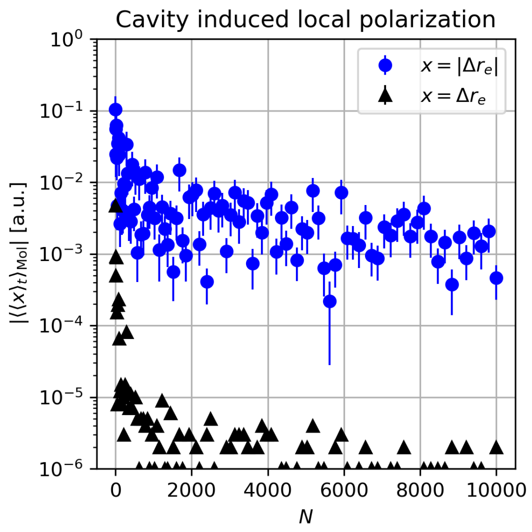


FIG. S2: Statistical evaluation of the electronic polarization $\Delta r_n(t) = \langle\hat{r}_n\rangle_\lambda - \langle\hat{r}_n\rangle_{\lambda=0}$ of the $n$-th molecule with respect to the bare molecule Shin-Metiu molecule in canonical equilibrium at $k_B T = 0.5$ [mH] for aligned molecules. The Rabi-splitting was kept constant when increasing the number of molecules by choosing a re-scaled $\lambda_\alpha(N) = 0.256/\sqrt{2N}$. By monitoring $|\Delta r_n|$ (blue dots), we observe qualitatively similar results comparing with the random oriented molecules. The simulations suggest a non-zero saturation of the cavity induced local polarizations in the large $N$ limit, where the standard deviations with respect to different molecules are displayed by vertical blue lines. At the same time, the total polarization of the ensemble, which is related to $\Delta r_n$ (black triangle), quickly approaches zero (exactly zero when averaging over longer time-scales), since the cavity cannot induce a non-zero ensemble polarization in thermal equilibrium. Consequently, our simulations suggest that cavity induced local strong coupling effects persist in the thermodynamic limit ($N \rightarrow \infty$) of a molecular ensemble under collective vibrational strong coupling. In other words, the self-consistent treatment is decisive to describe ground-state polaritonic chemistry accurately for collectively coupled molecular ensembles.

Supplementary simulation results for the vibrational absorption spectra of the molecular ensembles are shown in Fig. S3.

Local energy modifications for the ensembles of neon atoms are shown in Fig. S4. Since atoms do not have a permanent dipole moment, the energy contribution of the cavity-induced photon displacement $\hat{\boldsymbol{D}}$ and the dipole-dipole interaction term $V_{dd}$ are exactly zero, see Fig. S4 b) and d). Only the local cavity-induced polarization, see (Fig. S4 c), give rise to a non-zero contribution to

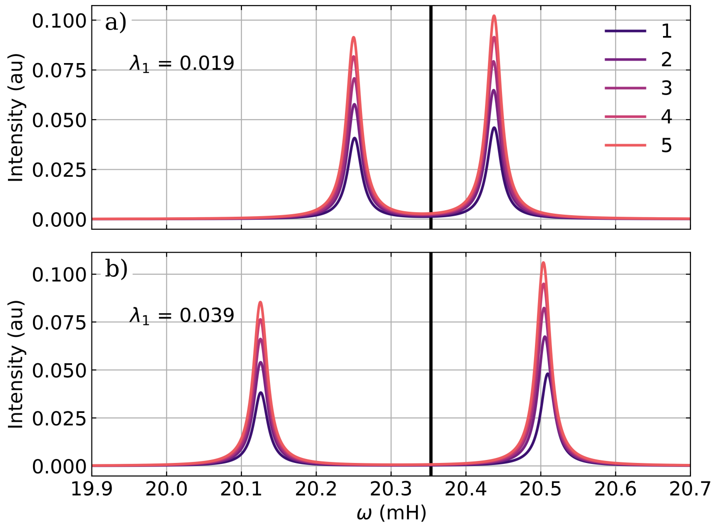


FIG. S3: Vibrational absorption spectra in the harmonic approximation for a few perfectly parallel aligned HF molecules under collective vibrational strong coupling. The number of molecules is color coded. The cavity is tuned to the first vibrational mode of the uncoupled HF molecule at $\omega_\alpha = 20.35$ [mH] (black vertical line) with unscaled coupling strength of a) $\lambda_1 = 0.019$ and b) $\lambda_1 = 0.039$.

the locale energy (see Fig. S4 a)) for the investigated ensemble sizes. However, this contribution decays with $\frac{1}{N}$ and is negligible in the large-$N$ limit.

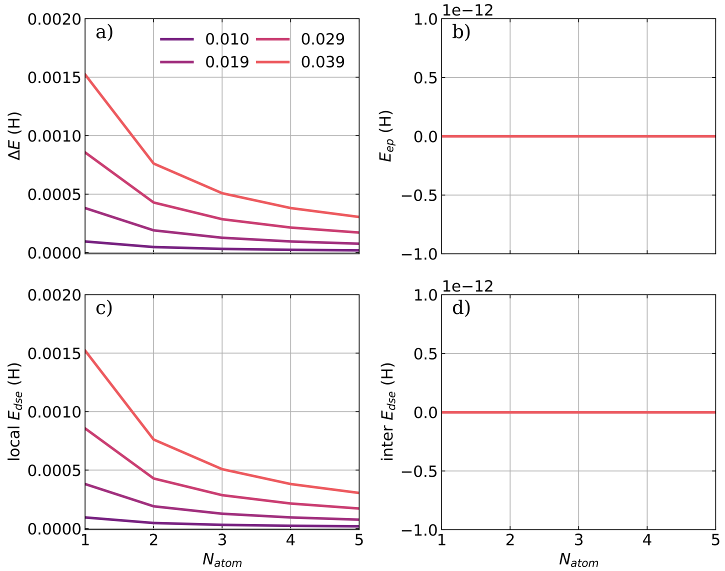


FIG. S4: Influence of the cavity interaction on an individual neon atom in ensembles of different size. a) The total energy change with respect to the cavity free situation, b) the local contribution of the cavity-induced photon displacement $E_{ep}$, c) the local cavity induced polarization contribution $E_{dse}$ and d) the dipole-dipole interaction term $V_{dd}$ as a function of $N_{atom}$. A cavity frequency $\omega_\alpha$ of 20.35 [mH] is used. The value of the unscaled coupling strength $\lambda_1$ is increased from 0.010 to 0.039 (color coded).

## REFERENCES


[1] J. Flick, H. Appel, M. Ruggenthaler, and A. Rubio, "Cavity born–oppenheimer approximation for correlated electron–nuclear-photon systems," J. Chem. Theory Comput. **13**, 1616–1625 (2017).

[2] D. Sidler, M. Ruggenthaler, C. Schaefer, E. Ronca, and A. Rubio, "A perspective on ab initio modeling of polaritonic chemistry: The role of non-equilibrium effects and quantum collectivity," J. Chem. Phys. **156** (2022), 10.1063/5.0094956, 230901.

[3] M. Ruggenthaler, D. Sidler, and A. Rubio, "Understanding polaritonic chemistry from ab initio quantum electrodynamics," Chem. Rev. **123**, 11191–11229 (2023).

[4] T. Schnappinger and M. Kowalewski, "Nonadiabatic wave packet dynamics with ab initio cavity-born-oppenheimer potential energy surfaces," J. Chem. Theory Comput. **19**, 460–471 (2023).

[5] E. W. Fischer and P. Saalfrank, "Vibro-Polaritonic chemistry beyond the cavity Born-Oppenheimer approximation and the role of electron-photon correlation," (2023),

arXiv:2305.11153 [physics.chem-ph].

[6]T. Schnappinger, D. Sidler, M. Ruggenthaler, A. Rubio, and M. Kowalewski, en“Cavity Born-Oppenheimer Hartree-Fock ansatz: Light-matter properties of strongly coupled molecular ensembles,” J. Phys. Chem. Lett. **14**, 8024–8033 (2023).

[7]R. M. Mazo, *Brownian motion: fluctuations, dynamics, and applications*, Vol. 112 (OUP Oxford, 2008).

[8]R. Marino, R. Eichhorn, and E. Aurell, “Entropy production of a brownian ellipsoid in the overdamped limit,” Phys. Rev. E **93**, 012132 (2016).

[9]G. Volpe and G. Volpe, “Simulation of a brownian particle in an optical trap,” American Journal of Physics **81**, 224–230 (2013).

[10]S. Shin and H. Metiu, “Nonadiabatic effects on the charge transfer rate constant: A numerical study of a simple model system,” J. Chem. Phys. **102**, 9285–9295 (1995).

[11]G. Albareda, H. Appel, I. Franco, A. Abedi, and A. Rubio, “Correlated electron-nuclear dynamics with conditional wave functions,” Phys. Rev. Lett. **113**, 083003 (2014).

[12]J. Yang, Q. Ou, Z. Pei, H. Wang, B. Weng, Z. Shuai, K. Mullen, and Y. Shao, “Quantum-electrodynamical time-dependent density functional theory within gaussian atomic basis,” J. Chem. Phys. **155**, 064107 (2021).

[13]G. Bussi and M. Parrinello, “Accurate sampling using langevin dynamics,” Phys. Rev. E **75**, 056707 (2007).

[14]P. H. Berens and K. R. Wilson, “Molecular dynamics and spectra. I. Diatomic rotation and vibration,” J. Chem. Phys. **74**, 4872–4882 (1981).

[15]R. B. Blackman and J. W. Tukey, “The measurement of power spectra from the point of view of communications engineering—part i,” Bell System Technical Journal **37**, 185–282 (1958).

[16]T. Schnappinger and M. Kowalewski, en“Ab initio Vibro-Polaritonic spectra in strongly coupled Cavity-Molecule systems,” J. Chem. Theory Comput. **19**, 9278–9289 (2023).

[17]D. G. Smith, L. A. Burns, D. A. Sirianni, D. R. Nascimento, A. Kumar, A. M. James, J. B. Schriber, T. Zhang, B. Zhang, A. S. Abbott, *et al.*, “Psi4numpy: An interactive quantum chemistry programming environment for reference implementations and rapid development,” J. Chem. Theory Comput. **14**, 3504–3511 (2018).

[18]D. G. Smith, L. A. Burns, A. C. Simmonett, R. M. Parrish, M. C. Schieber, R. Galvelis, P. Kraus, H. Kruse, R. Di Remigio, A. Alenaizan, *et al.*, “Psi4 1.4: Open-source software for high-throughput quantum chemistry,” J. Chem. Phys. **152** (2020).

[19]R. A. Kendall, T. H. Dunning,  and R. J. Harrison, “Electron affinities of the first-row atoms revisited. systematic basis sets and wave functions,” J. Chem. Phys. **96**, 6796–6806 (1992).

[20]M. Kowalewski and P. Seeber, “Sustainable packaging of quantum chemistry software with the nix package manager,” Int. J. Quant. Chem. **122**, e26872 (2022).
