# Supplementary material for: Unraveling a Cavity-Induced Molecular Polarization Mechanism from Collective Vibrational Strong Coupling
Source: J Phys Chem Lett. 2024 May 8;15(19):5208–14. doi: 10.1021/acs.jpclett.4c00913 (PMC11103705; doi:10.1021/acs.jpclett.4c00913)
Supplement: Supplementary file 2 — jz4c00913_si_002.pdf [file jz4c00913_si_002.pdf]

jz-2024-009139.R1

Name: Peer Review Information for "Unraveling a Cavity Induced Molecular Polarization Mechanism From Collective Vibrational Strong Coupling"

First Round of Reviewer Comments

Reviewer: 1

Comments to the Author

In this work, Cavity HF theory is used to investigate the origin of the collective effect in Vibrational strong coupling effects. The finding is very interesting and potentially insightful, which is the collective VSC coupling can alter individual molecules strongly for localized hotspots, similar to the spin glass phase. This work thus provides very valuable insights into the VSC effects. It is also novel to point out that I think such insights are extremely valuable to answer the mysteries of the VSC effects and help the field of polariton chemistry move forward.

I thus enthusiastically support this work to be published in JPCL. I only have a few questions that the authors could consider to address

(1) Given the current theory, would one be able to connect the N=1 rate theory of the VSC effects? For example, those in [J. Chem. Phys. 159, 084104 (2023)], [doi.org/10.1515/nanoph-2023-0685], [10.26434/chemrxiv-2024-sl6lt]

(2) Why nuclear dipoles are not considered in the dipole-dipole interactions (Eq 4), are they considered somewhere else?

(3) it will be great to visualize these hotspots in terms of molecular configurations, so we understand more about those hotspots

(4) When reporting the results, it might be more clear to use cm^-1 as this is the commonly used energy unit in VSC.

Author's Response to Peer Review Comments:

MAX PLANCK INSTITUTE FOR THE STRUCTURE

AND DYNAMICS OF MATTER

Theory Department

MPSD, Luruper Chaussee 149, D-22761 Hamburg

Editorial Board

Journal of Physical Chemistry Letters

Dominik Sidler
Luruper Chaussee 149
D-22761 Hamburg

Phone: +49 40 8998 88340
dsidler@mpsd.mpg.de

Hamburg, May 1, 2024

Dear Editor

Please find below the detailed response to the referees.

Yours sincerely,

Dominik Sidler, on behalf of all authors.

Reviewer 1:

In this work, Cavity HF theory is used to investigate the origin of the collective effect in Vibrational strong coupling effects. The finding is very interesting and potentially insightful, which is the collective VSC coupling can alter individual molecules strongly for localized hotspots, similar to the spin glass phase. This work thus provides very valuable insights into the VSC effects. It is also novel to point out that I think such insights are extremely valuable to answer the mysteries of the VSC effects and help the field of polariton chemistry move forward. I thus enthusiastically support this work to be published in JPCL. I only have a few questions that the authors could consider to address

Response:

We thank the referee very much for his/her very positive and encouraging feedback. We have addressed all the suggested changes in the revised version of the manuscript, which significantly improved the quality and clarity of our work. See detailed feedback below.

Given the current theory, would one be able to connect the N=1 rate theory of the VSC effects? For example, those in [J. Chem. Phys. 159, 084104 (2023)], [doi.org/10.1515/nanoph-20230685], [10.26434/chemrxiv-2024-sl6lt]

Response:

You raised a very interesting and relevant point. Indeed, it is tempting to ask if one can connect our collective induced local effects to local rate theories derived for $N = 1$. At the moment, we think our understanding of the thermodynamic consequences of the polarizaton glass phase and the physics of the hotspots (see reply 3 below) is not sufficient to answer this question. To better understand the chemical reaction rate properties, we intend to investigate the Shin-Metiu setup in that context in the future. This will hopefully provide insights on how polaritonic rates can be derived from $N = 1$ simulation results, which is of utter relevance to reach for computational reaction rate predictions of complex molecules. We have highlighted this aspect now more thoroughly in the outlook of our manuscript.

(2) Why nuclear dipoles are not considered in the dipole-dipole interactions (Eq 4), are they considered somewhere else?

Response:

Thanks for raising this aspect. The nuclear dipole-dipole interactions do not appear in the dressed electronic problem when applying the cavity-Born-Oppenheimer partitioning, i.e.,

---

when separating the electrons from the (in our case) classical nuclear-photon degrees of freedom. We have now improved the description of Eq. (4) to avoid confusion and explicitly referred to the SI, where the partitioning is made explicit.

(3) it will be great to visualize these hotspots in terms of molecular configurations, so we understand more about those hotspots

Response:

You point to a very interesting, but non-trivial aspect of our work. We deliberately did not provide a quantitative or pictorial physical description of the hotspots. The reason is that reaching for a physical meaningful picture / understanding will be a project on its own, since it will involve the analysis of all the implied time-scales of the polarization glass phase. For example, on what time-scales does a hot-spot form and persist and how is it related to the vibrational time-scale as well as the corresponding local nuclear configuration and collective distribution? We are afraid that simply selecting a strongly polarized molecule at a single time-step does not provide meaningful physical insights without understanding the statistical context and in the worst case such a selective visualization might even be misleading. However, providing the statistical and thermodynamic context will be a highly non-trivial task, when anticipating properties from well known spin glass theory (i.e., breaking of fluctuation-dissipation, frustration with different implied time-scales and dynamics). Therefore, we keep the detailed study of the physics of hotspots for future work, which we intend to pursue in parallel with the reaction rate aspects. However, we now raise these delicate and important open aspects of the hotspots more prominently in the the manuscript.

(4) When reporting the results, it might be more clear to use cm^{-1} as this is the commonly used energy unit in VSC.

Response:

We agree that cm^{-1} might be a more appropriate choice for chemical applications. However, depending on the scientific background different unit choices might seem more natural, which we believe is very common due to the very interdisciplinary nature of the emergent field of polaritonic chemistry. For this reason, we kept our choice and hope that you agree with us. Notice further that while our main findings, derived from the Shin-Metiu model, unravel a qualitatively physical mechanism in the vibrational strong coupling regime, it does not allow to make quantitative statements for real molecular ensembles. Thus for our setup the unit choice may also be arbitrary, provided that the ensemble properties are compliant with the involved assumptions (e.g., VSC and cavity BOA applicable).
